# An adaptive geometry regulation strategy for 3D graphene materials: towards advanced hybrid photocatalysts†
†Electronic supplementary information (ESI) available. See DOI: 10.1039/c8sc03679a


**DOI:** 10.1039/c8sc03679a

**Published:** 2018-09-24

**Authors:** Xiuqiang Xie, Nan Zhang, Zi-Rong Tang, Yi-Jun Xu

**Affiliations:** a State Key Laboratory of Photocatalysis on Energy and Environment , College of Chemistry , Fuzhou University , Fuzhou 350116 , P. R. China . Email: yjxu@fzu.edu.cn; b College of Chemistry , New Campus , Fuzhou University , Fuzhou 350116 , P. R. China

## Abstract

Three-dimensional graphene (3DG) is promising for constructing monolithic photocatalysts for solar energy conversion. However, the structure-associated light-shielding effect and the intricate porous architecture of 3DG result in intrinsic limitations in light penetration and mass transfer over 3DG supported hybrids, which restricts their photocatalytic efficiency. Here, taking 3DG–organic hybrids as examples, we report a geometry regulation strategy to minimize such structural restrictions, which not only favors the interaction between light and the photoactive component, but also facilitates reactant adsorption over the 3DG–organic hybrids, thereby cooperatively boosting their photoactivity. Such an adaptive geometry regulation strategy is expected to guide the rational utilization of 3DG to construct high-performance hybrids for photoredox catalysis.

## Abstract 2

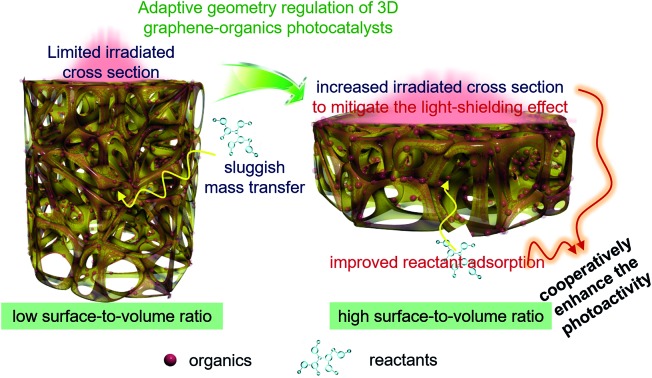
Geometry regulation of 3D graphene-based photocatalysts by increasing the surface-to-volume ratio to concurrently promote light–photoactive material interactions and improve reactant adsorption towards enhanced performance.

## Introduction

Monolithic photocatalysts have attracted significant attention in recent years due to their simple recyclability for practical applications. With considerable advances brought about by two-dimensional graphene (2DG) in the area of solar energy conversion,^1^–^12^ three-dimensional graphene (3DG) materials assembled from 2DG have emerged as a paradigm in the design of monolithic hybrid photocatalysts, which not only features convenient recycling, but also affords (1) multidimensional electron transfer pathways to promote the spatial separation/transfer of photogenerated charge carriers;^13^–^15^ and (2) hierarchical porous structures to substantially inhibit the aggregation of subunits, thus exposing more accessible surface-active sites.^14^ Hitherto, a variety of photoactive materials have been integrated with 3DG,^14^,^16^–^23^ and they have promising prospects in a wide range of photoredox applications including water splitting,^22^,^24^,^25^ selective chemical synthesis,^14^,^26^–^29^ and water remediation.^30^–^34^

3DG itself has intrinsic restrictions for solar energy conversion despite certain attractive advantages of 3DG in the preparation of efficient macroscopic assemblies for photoredox catalysis. The most prominent one relates to a shielding effect, which leads to a trade-off between the formation of a 3DG network and the efficient light–photoactive material interaction. More specifically, the formation of a 3DG network normally requires a critical high concentration of graphene, which inevitably reduces the light penetration depth into 3DG-based hybrids.^7^,^9^,^35^,^36^ Such a situation compromises light–photoactive material interactions and thus the performance of 3DG supported hybrid photocatalysts deteriorates. In addition, the channels of 3DG-based photocatalysts are typically disordered and present intricate reactant diffusion pathways, although the prevented aggregation of graphene by the hierarchical assembly into 3D porous structures increases the likelihood of reactants interacting with the surface active sites.^37^ This results in sluggish internal mass transfer of reactants, restricting the overall reactant adsorption and the efficiency of photocatalytic surface reactions. Considering the key influences of the above issues on the photoactivity of 3DG-based hybrids, studies on how to mitigate these intrinsic structure-associated limitations for the rational utilization of 3DG to construct efficient monolithic hybrids for photoredox catalysis are essential but still lacking in the literature.

Herein, we report a simple and efficient adaptive geometry modulation strategy to simultaneously mitigate the light-shielding effect and improve reactant adsorption on 3DG-based photocatalysts. We selected organic dyes (Eosin Y (EY) and Rose Bengal (RB)) as model photoactive components, which can be homogeneously immobilized onto the scaffold of 3DG to synthesize the supported 3DG–organic hybrids.^14^ By modulating the geometry of 3DG, an increased surface-to-volume ratio of 3DG–organics was achieved, which not only increases the light irradiated cross section to enhance the light–photoactive material interaction and boost the generation of photogenerated charge carriers, but also favors the reactant adsorption by minimizing the ratio of the intricate internal porous structure. In turn, these merits reduce the intrinsic structural restrictions of 3DG supported hybrids, thereby cooperatively enhancing the photocatalytic efficiency. This work highlights the importance of rational design and utilization of 3DG scaffolds to prepare advanced monolithic hybrid photocatalysts for solar energy conversion.

## Results and discussion

**Fig. 1:**
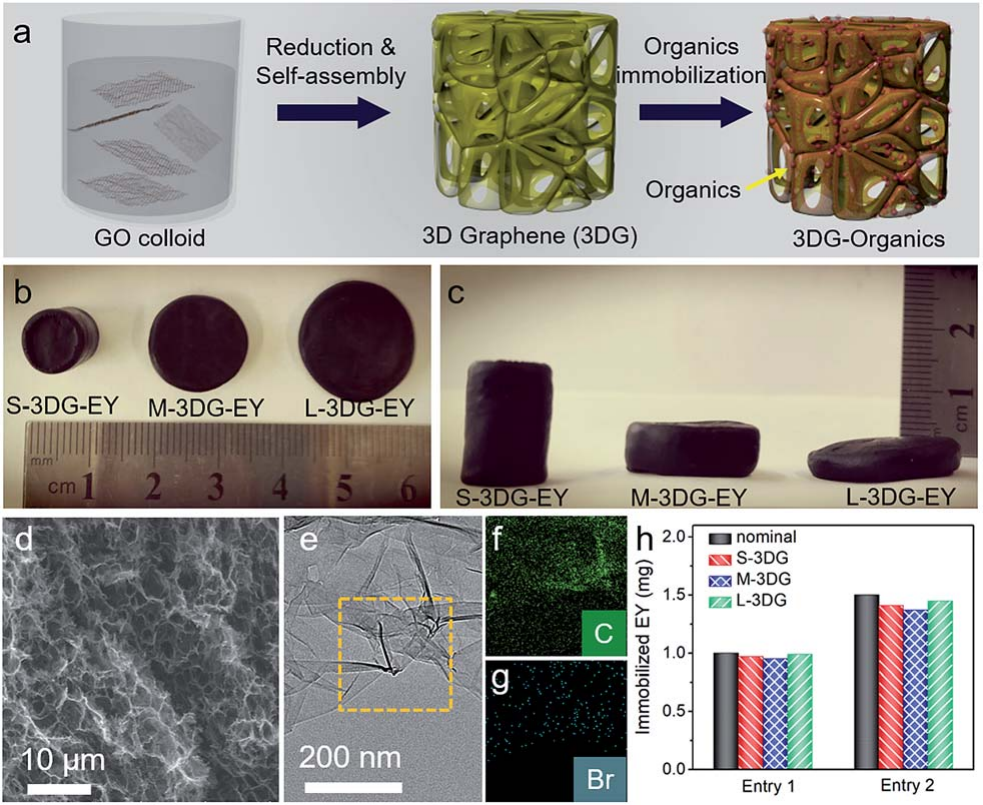
Schematic for the preparation of the 3DG–organic photocatalysts (a). Top-view (b) and side-view (c) photos of the as-prepared 3DG–EY hybrids with different geometries. SEM image (d), TEM image (e) and elemental mapping (f and g) of the L-3DG–EY hybrids in the selected area indicated by the yellow rectangle in (e). Amount of EY immobilized over S-3DG, M-3DG and L-3DG (h).

The preparation of 3DG–organic photocatalysts involves the construction of 3DG followed by immobilization of organic dyes as photoactive components (Fig. 1a). Notably, the self-assembly process of graphene oxide (GO) into 3DG is isotropic, and the geometric parameters of the resulting 3DG critically depend on the geometry of the GO colloid.^38^ Consequently, the geometry of 3DG and the resulting 3DG–organics can be feasibly regulated by simply using different containers while keeping the total volume of the GO precursor unchanged. In the present study, glass cylinders with capacities of 40 mL, 50 mL, and 100 mL have been used to prepare 3DG with different geometries, denoted as S-3DG, M-3DG, and L-3DG, respectively. The *I*_D_/*I*_G_ ratios of S-3DG, M-3DG, and L-3DG are 1.51, 1.53, and 1.51, respectively, indicating that these 3DG scaffolds have identical defect densities (Fig. S1†). The organic dyes were immobilized on 3DG *via* a hydrothermal step by means of π–π interactions between delocalized π-conjugated bonds of the organic molecules (the structures of EY and RB are shown in Fig. S2†) and the large π-conjugated structure of graphene. This two-step strategy utilizing pre-synthesized 3DG to prepare 3DG–organics can avoid the interference arising from immobilization of organics in the self-assembly process of 3DG and enable an independent study on the influence of geometry effects of 3DG on the photocatalytic performance of 3DG-based composites. Fig. 1b and c show the macroscopic features of the resultant 3DG–EY hybrids with varied geometries, which progressively changed from a cylindrical to an oblate-type shape. In addition, they are typically black in color due to the high concentration of graphene (similar to other reported 3DG-based photocatalysts), which inevitably blocks light penetration into the internal area and restricts the pre-requisite light–photoactive material interaction for efficient generation of charge carriers. Table S1† summarizes all geometry parameters, revealing that the cross-sectional areas and surface-to-volume ratios of S-3DG–EY, M-3DG–EY and L-3DG–EY increase gradually.

Fig. 1d shows the scanning electron microscopy (SEM) image of the L-3DG–EY hybrids. The cross-linking of the graphene generated 3D networks with hierarchical macropores in the range of several micrometers can be clearly visualized. The disordered channels could potentially limit the rapid diffusion of reactants, which restricts the effective utilization of the internal surface of the 3DG–organic photocatalysts, as discussed later. S-3DG–EY and M-3DG–EY hybrids with different geometries have similar porous networks (Fig. S3†). Fig. 1e shows the transmission electron microscopy (TEM) image of the L-3DG–EY hybrids, exhibiting a wrinkled morphology consistent with SEM observation. Notably, no typical structure of EY has been observed from SEM and TEM images, suggesting the high dispersion of molecular EY in the framework of graphene through π–π interactions. The composition of 3DG–EY has been investigated by energy-dispersive X-ray (EDX) analysis. The results (Fig. S4†) pointed to the coexistence of C, O, and Br (typical elements of EY). The elemental mapping results shown in Fig. 1f and g further demonstrate that C and Br are homogenously distributed (Fig. 1e), implying the uniform distribution of organic EY throughout 3DG. From Table S2† it can be seen that the maximum amounts of EY/RB immobilized over 3DG with different geometries are identical, reaching ∼2.6 mg. 3DG–EY hybrids with nominal EY loading amounts of 1.0 and 1.5 mg have been studied, and are denoted as 3DG–EY-1.0 and 3DG–EY-1.5, respectively. Fig. 1h demonstrates that the amounts of EY immobilized over S-3DG, M-3DG and L-3DG are very close to the nominal values. The identical mass of the 3DG scaffold and immobilized photoactive material of EY together with the similar porous structure further offers the basis for a reasonable investigation of the geometry effect on the photocatalytic activity of the 3DG–EY hybrids.

**Fig. 2:**
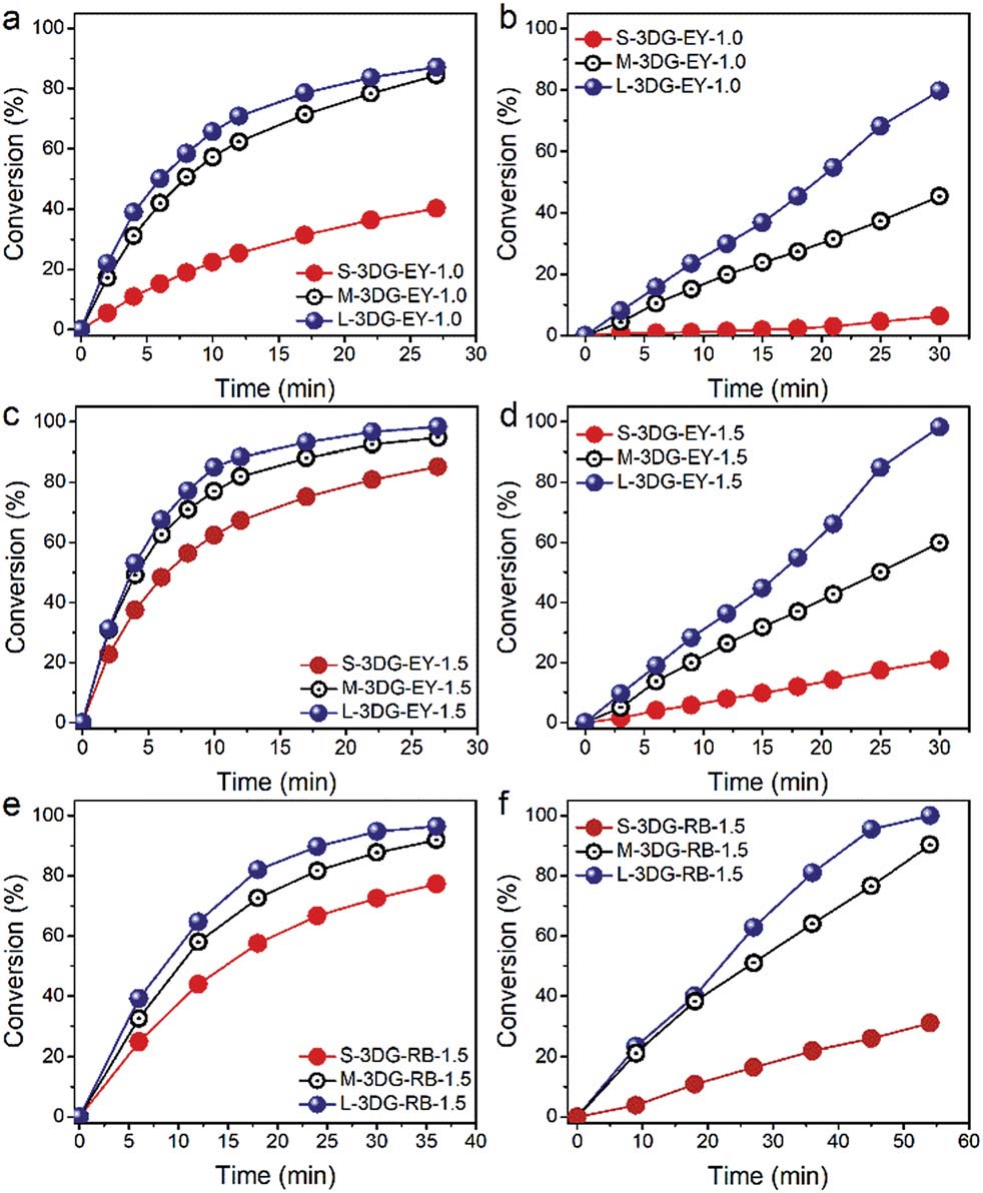
Time-online profiles of photocatalytic hydrogenation of 4-NA (a, c, and e) and photoreduction of Cr(vi) (b, d, and f) over different 3DG-based photocatalysts under visible light irradiation (≥420 nm).

The photocatalytic activity of the as-prepared 3DG–EY hybrids has been evaluated by the photocatalytic hydrogenation of 4-nitroaniline (4-NA), an important intermediate for the synthesis of rubber antioxidants and aramid textile fiber in the chemical industry.^39^ The visible light irradiation direction is chosen to be vertical to the cross-sectional area of the 3DG–EY hybrids (as indicated in Fig. S5†), which ensures that the incident light power density is constant and affords a rational investigation of the geometry effect on the photoactivity of the 3DG-based hybrids. As shown in Fig. 2a, the conversion of 4-NA over S-3DG–EY-1.0 reaches 40% after 27 min. 4-NA was converted to the corresponding amine compound of 4-phenylenediamine (4-PDA) by photogenerated electrons (Fig. S6†). When the geometry of the 3DG–EY hybrids progressively changes, the photoactivity improved remarkably in the systems, reaching a 4-NA conversion of 84% and 87% over M-3DG–EY-1.0 and L-3DG–EY-1.0 under identical reaction conditions, respectively. The analogous enhanced performances of the 3DG–EY hybrids by a simple geometry regulation of 3DG have also been observed in the photocatalytic reduction of Cr(vi), following the order L-3DG–EY-1.0 > M-3DG–EY-1.0 > S-3DG–EY-1.0. The optimum L-3DG–EY-1.0 can efficiently convert 80% of Cr(vi) under visible light irradiation after 30 min (Fig. 2b). A higher EY loading amount of 1.5 mg further increased the photoactivity towards the conversion of 4-NA (Fig. 2c) and reduction of Cr(vi) (Fig. 2d), suggesting that EY is the photoactive ingredient in these monolithic hybrid photocatalysts. In addition, the trend for the photoactivity enhancement has been found to be the same, following the order L-3DG–EY-1.5 > M-3DG–EY-1.5 > S-3DG–EY-1.5.

To verify whether the geometry modulation is generic to enhance the performance of 3DG-based photocatalysts, we have further prepared a series of 3DG-based photocatalysts following the same synthetic procedure by replacing EY with another organic dye, RB. Similarly, the optimum L-3DG immobilized with a nominal RB amount of 1.5 mg (L-3DG–RB-1.5) exhibited enhanced visible-light photoactivity as compared to its S-3DG–RB-1.5 and M-3DG–RB-1.5 counterparts for both the photocatalytic transformation of 4-NA (Fig. 2e) and reduction of Cr(vi) (Fig. 2f). Notably, no photocatalytic conversion of 4-NA or Cr(vi) has been observed over S-3DG, M-3DG, and L-3DG, suggesting that the organic sensitizers EY and RB are the photoactive components in our system (Fig. S7†). These results clearly demonstrate the versatility of this simple yet efficient geometry optimization of the 3DG substrate for improving the photoactivity of the 3DG–organic hybrids.

**Fig. 3:**
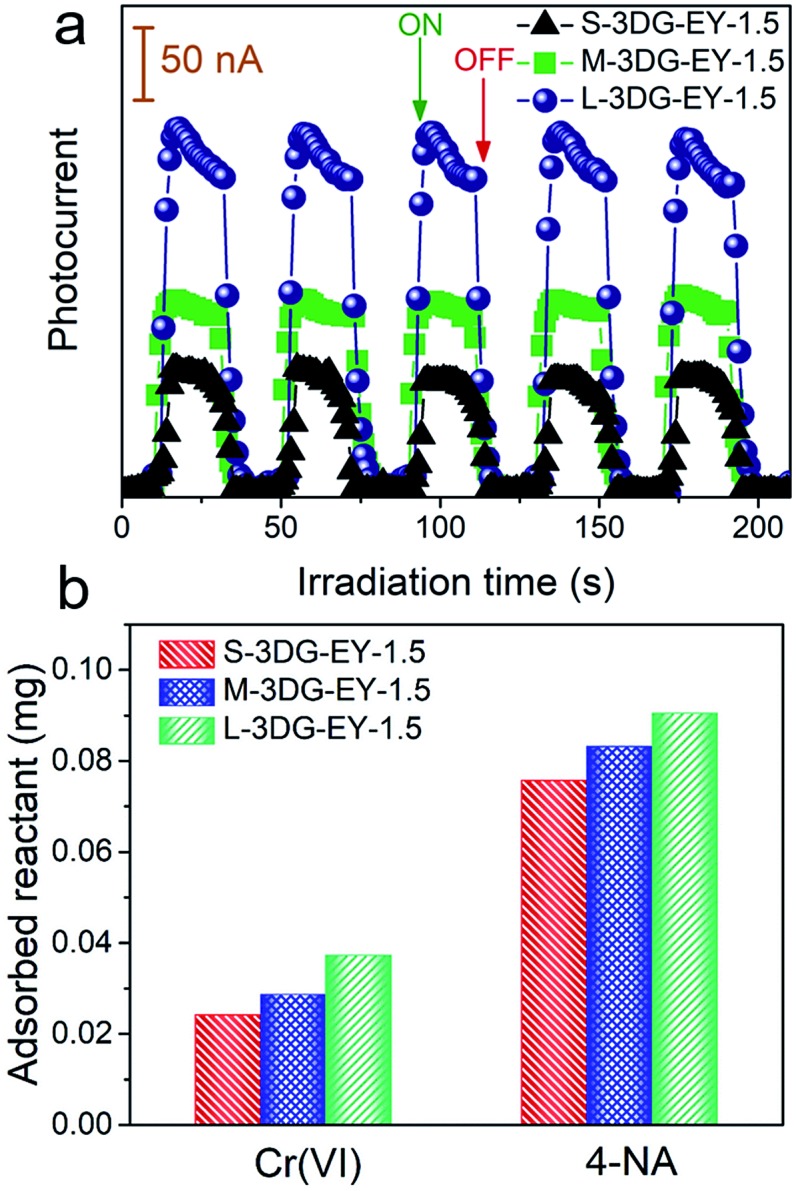
Transient photocurrent spectra of S-3DG–EY-1.5, M-3DG–EY-1.5 and L-3DG–EY-1.5 under visible light irradiation (≥420 nm) (a). Adsorption properties of S-3DG–EY-1.5, M-3DG–EY-1.5 and L-3DG–EY-1.5 towards Cr(vi) and 4-NA (b).

As described above, the L-3DG-based hybrids with the highest surface-to-volume ratio show the best visible-light photoactivity in all experiments. To get a deeper insight into the geometry effect on the photoactivity enhancement of the 3DG–organic hybrids, combined investigations were conducted. The results from Fig. 3a indicate that the transient photocurrent increases in the order of L-3DG–EY-1.5 > M-3DG–EY-1.5 > S-3DG–EY-1.5 under visible light irradiation. Since the electrical conductivities of L-3DG–EY-1.5, M-3DG–EY-1.5 and S-3DG–EY-1.5 are similar (Fig. S8†), it can be deduced that the difference in photocurrent response over the samples correlates with the excitation efficiency of EY rather than the separation and transfer of charge carriers photogenerated from the excitation of organic dyes in the current system. In other words, the photocurrent transient response results suggest that the light-shielding effect of graphene is effectively mitigated through increasing the cross-sectional area directly exposed to the light irradiation. Fig. 3b shows the adsorption properties of S-3DG–EY-1.5, M-3DG–EY-1.5 and L-3DG–EY-1.5 towards Cr(vi) and 4-NA in the dark. The adsorbed amount of Cr(vi) increases in the order of L-3DG–EY-1.5 > M-3DG–EY-1.5 > S-3DG–EY-1.5. A similar trend has been observed for the adsorption of 4-NA. Notably, the adsorption capability of these photocatalysts follows the same order as their surface-to-volume ratio, whereas they have similar BET surface areas (Table S3†). These results indicate that the internal surfaces of 3DG cannot be fully utilized for reactant adsorption due to the sluggish mass transfer originating from the intricate porous structure. Comparably, the increased surface-to-volume ratio favors the adsorption of 4-NA by maximizing the external surface of the 3DG–organic hybrids.

**Fig. 4:**
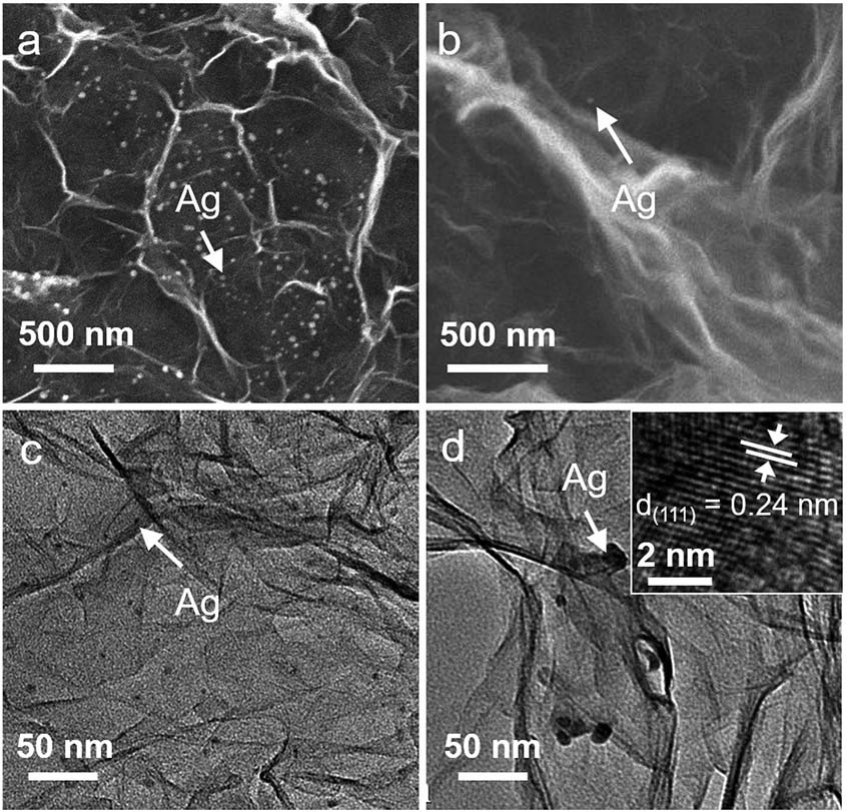
SEM and TEM images of the external surface (a and c) and internal surface (b and d) of L-3DG–EY-1.5 after the photocatalytic reduction of Ag^+^. The inset in d shows the HRTEM image of a Ag nanoparticle.

In the following, we further investigated how the surface-to-volume ratio of 3DG affects the photoactivity of the 3DG–organic hybrids. By utilizing the reaction of Ag^+^ + e^–^ → Ag^0^ performed over 3DG–organics under visible light irradiation, the spatial distribution of the photoreduction reactive sites of the 3DG-based photocatalysts has been investigated. The SEM images (Fig. 4a and b) clearly show that Ag nanoparticles are mainly deposited on the external surface of L-3DG–EY-1.5 while only few Ag nanoparticles can be observed on the internal surface. TEM analysis has also been conducted to further check the deposition of Ag nanoparticles, with results in good agreement with SEM observations (the density of Ag nanoparticles is obviously higher on the external surface than on the internal surface, Fig. 4c and d). The inset in Fig. 4d shows the HRTEM image of a Ag nanoparticle, from which a lattice spacing of 0.24 nm can be clearly seen, corresponding to the (111) crystal plane of Ag. The probing of the spatial reactive sites by photocatalytic reduction of Ag^+^ suggests that the primary reactive sites are located on the external surface of the 3DG-based monolithic photocatalysts. Moreover, this result indicates that although the electrons photogenerated from the excitation of EY can transfer throughout the conductive network of 3DG to drive the reduction of Ag^+^ on the internal surface of 3DG–EY, the mass transfer of Ag^+^ to the interstitial areas is the rate-limiting step due to the intricate diffusion channels, resulting in the limited deposition of Ag on the internal surface. Consequently, it can be concluded that the increased surface-to-volume ratio by geometry regulation is beneficial for reactant adsorption by minimizing the interstitial areas, thereby enhancing the photocatalytic activity of the 3DG–organic hybrids.

In addition to using the 3DG–EY composites for photocatalytic reactions, we conducted photocatalytic H_2_ evolution reactions in EY solutions over 3DGs with different geometries. Before the photocatalytic reactions, Pt particles were loaded over 3DGs as the low overpotential cocatalyst to facilitate H_2_ evolution.^40^ The photocatalytic H_2_ evolution experiments ensure that the light irradiation areas are identical over different 3DG-based systems. As shown in Fig. S9,† the H_2_ evolution rate increases in the order of L-3DG > M-3DG > S-3DG, providing direct evidence to support the view that the surface reactive sites improved by the adaptive geometry regulation of the 3DG scaffold contribute to enhancing the photocatalytic activity of the 3DG-based photocatalysts.

**Fig. 5:**
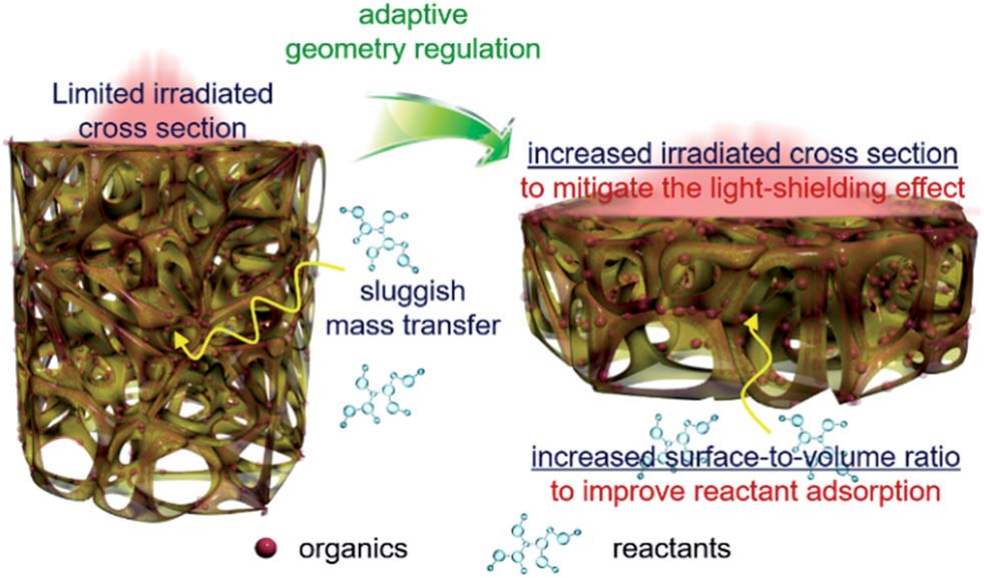
Schematic illustration of the adaptive geometry regulation to mitigate the light-shielding effect and improve mass transfer towards enhancing the photoactivity of the 3DG–organic hybrids.

Based on the above results, geometry optimization endows the 3DG–organic hybrids with improved photocatalytic efficiency from two cooperative aspects. The light-shielding effect of graphene leads to a limited light penetration into the interstitial part of the 3DG–organic photocatalysts (Fig. 5). The cylinder-shaped 3DG-based photocatalysts with a low surface-to-volume ratio have a small irradiated cross section, thereby limiting the excitation of the photoactive components. By adaptive geometry optimization, the irradiated cross-sectional area is increased, which mitigates the light-shielding effect of graphene for more efficient generation of charge carriers in the photoactive materials. In addition, the higher surface-to-volume ratio of 3DG–organics is favorable for the reactant adsorption and thus photocatalytic surface reactions, considering that the internal mass transfer is sluggish. Geometry-regulated 3DG with a high surface-to-volume ratio combines these favorable factors, which improves the formation and utilization of charge carriers photogenerated in the photoactive components, and cooperatively enhances the photocatalytic performance of the 3DG–organic monolithic hybrids as a consequence.

From Fig. S10† it can be seen that no obvious desorption of either EY or RB was observed in a pH range of 1.0–12.5, suggesting the good desorption resistance of the immobilized dyes due to the favorable π–π interactions between the organic molecules and the 3DG scaffolds. Taking L-3DG–EY-1.5 as an example, we have evaluated the recyclability in the photocatalytic hydrogenation of 4-NA. The good mechanical properties of the 3DG–organic photocatalysts (Fig. S11†) indicate that they can be easily recycled after the photocatalysis by simply using a tweezer (Fig. S12†), which is of practical significance for various applications. As shown in Fig. S13,† L-3DG–EY-1.5 can continuously transform 4-NA into 4-PDA under visible light irradiation. A noticeable photoactivity decrease can be observed, which can be ascribed to the consumption of EY during the photocatalytic process as commonly observed in dye-sensitized photocatalytic systems.^41^ Nevertheless, the regeneration of the 3DG–organic photocatalysts was successfully accomplished by re-immobilizing EY over reused L-3DG–EY-1.5 (Fig. S13†).

## Conclusions

In summary, advanced 3DG–organic photocatalysts have been prepared by a simple and efficient two-step strategy, which involves pre-synthesis of 3DG and sequential immobilization of organics as photoactive materials. A simple, adaptive geometry regulation protocol to increase the surface-to-volume ratio of the 3DG scaffold enabled the photoactivity enhancement of the 3DG–organic photocatalysts due to the effects of geometry regulation of 3DG–organics that (1) minimize the light-shielding effect of graphene by increasing the light–photoactive material interaction and (2) improve the reactant adsorption for photocatalytic surface reactions by generating a high surface-to-volume ratio. This study highlights the effectiveness and significance of regulating the geometry of 3DG to mitigate its intrinsic limitations for the design of advanced photocatalytic 3DG-based hybrids, which can pave the way for rational utilization of 3DG as an attractive scaffold to design more efficient and easily recyclable photocatalysts for solar energy conversion.

## Experimental

### Materials

Hydrochloric acid (HCl), concentrated sulfuric acid (H_2_SO_4_, 98%), potassium permanganate (KMnO_4_), hydrogen peroxide (H_2_O_2_, 30%), silver nitrate (AgNO_3_), triethanolamine (TEOA), EY, and RB were all obtained from Sinopharm Chemical Reagent Co., Ltd. (Shanghai, China). Graphite powder was supplied by Qingdao Zhongtian Company, China. All reagents were used as received without further purification. The deionized (DI) water used in the experiment was from local sources.

### Preparation of 3DG with different geometries

Graphene oxide (GO) was synthesized from natural graphite powder by a modified Hummers method.^42^–^45^ To prepare 3DG, 2 mL of NaHSO_3_ solution (60 mg mL^–1^) was added dropwise into 15 mL of GO suspension (2 mg mL^–1^). After stirring at room temperature for 5 min, the homogeneous mixture was kept in an oven at 70 °C for 12 h. Glass cylinders with capacities of 40 mL, 50 mL, and 100 mL were used to prepare S-3DG, M-3DG, and L-3DG, respectively. After being cooled down to room temperature naturally, the 3DG was rinsed with DI water for 24 h.^38^

### Immobilization of organic photoactive materials

The organics (EY or RB) were first dissolved in DI water to give a concentration of 0.5 mg mL^–1^. To immobilize 1 mg of organics, 2 mL of the solution was added to 18 mL of DI water and stirred for 5 min. The mixture was transferred into a 25 mL Teflon-lined autoclave, into which the as-prepared 3DG was added. After hydrothermal treatment at 120 °C for 12 h, the 3DG–organic composite was taken out with a pair of tweezers and washed several times with DI water. The immobilization of 1.5 mg of organics was performed by a similar procedure except that 3 mL of organic solution (0.5 mg mL^–1^) was mixed with 17 mL of DI water. The amount of immobilized organics was determined by the concentration change of the organics probed by an ultraviolet-visible light (UV-vis) spectrophotometer.

### Materials characterization

The morphology of the samples was determined by field emission scanning electron microscopy (FE-SEM) on an FEI Nova NANOSEM 230 spectrophotometer. Transmission electron microscopy (TEM) images and elemental mapping results were obtained using a JEOL model JEM 2010 EX instrument at an acceleration voltage of 200 kV. Micromeritics ASAP 3020 equipment was used to determine the nitrogen adsorption–desorption isotherms and the Brunauer–Emmett–Teller (BET) surface areas at 77 K. The samples were degassed at 180 °C for 5 h and then analyzed at 77 K. The relative pressure (*P*/*P*_0_) range used for the calculation of the BET surface area was from 0.05 to 0.35. The electrical conductivity measurement of 3DG with different geometries was performed by measuring the resistivity at a pressure of 12 MPa using a four-point probe technique on a ST2722 (Suzhou Jingge Electronic Co., China) power resistivity tester.

Photoelectrochemical measurements were performed in a homemade three electrode quartz cell using an Autolab electrochemical workstation. A Pt plate was used as the counter electrode, and a Ag/AgCl electrode was used as the reference electrode. The working electrode was prepared on fluorine-doped tin oxide (FTO) glass cleaned by ultrasonication in ethanol for 30 min and dried at 80 °C. The as-prepared 3DG–EY was directly stuck onto FTO glass using a piece of carbon tape and the uncoated part of the FTO was isolated with epoxy resin. The photocurrent measurement was carried out and the electrolyte was 0.2 M aqueous Na_2_SO_4_ solution (pH = 6.8). The visible light irradiation source was a 300 W Xe arc lamp system equipped with a UV-CUT filter (*λ* ≥ 420 nm) with the incident light being vertical to the cross-sectional area of the 3DG–EY composites.

### Photocatalytic activity tests

In a typical photocatalytic reaction, a 300 W Xe arc lamp (PLS-SXE 300, Beijing Perfect light Co., Ltd.) with a UV-CUT filter (*λ* ≥ 420 nm) was used as the irradiation source. The 3DG–organic photocatalysts and 60 μL of triethanolamine (TEOA, sacrificial agent) were added into 60 mL of a 4-nitroaniline solution (10 mg L^–1^) or Cr(vi) solution (10 mg L^–1^) in a quartz vial. Before visible light illumination, the above suspension was kept in the dark for 1 h to reach adsorption–desorption equilibrium. During the process of the reaction, 3 mL of sample solution was collected at a certain time interval and analyzed on a Varian ultraviolet-visible light (UV-vis) spectrophotometer (Cary-50, Varian Co.). The whole experimental process was conducted under N_2_ purging at a flow rate of 80 mL min^–1^. The incident light power density was 383 mW cm^–2^ and the irradiation direction was vertical to the cross-sectional area of the 3DG–organic composites.

Before photocatalytic H_2_ evolution tests, 3DGs were first loaded with Pt by a photodeposition method under UV-vis irradiation. Typically, 3DG was immersed in a mixture of DI water (20 mL), ethanol (1 mL), H_2_PtCl_6_ aqueous solution (0.615 mM, 2.5 mL), and EY (1.5 mg). The system was then purged with N_2_ for 30 min. After UV-vis irradiation for 2 h under N_2_ purging, Pt-loaded 3DG was taken from the mixture and washed with DI water. The nominal Pt loading ratio is 1 wt% over 3DG. For each photocatalytic H_2_ evolution test, the Pt-loaded 3DG was then added to 10 mL of triethanolamine (TEOA) aqueous solution (10 vol%), to which 1.5 mg of EY was added. The reaction solution was purged with N_2_ to remove air prior to irradiation. The visible light irradiation source was a 300 W Xe arc lamp system equipped with a UV-CUT filter (*λ* ≥ 420 nm). The incident light power density was 383 mW cm^–2^. The photocatalytic reaction was typically performed for 4 h, and 1 mL of reactive gas was taken from the reactor with a syringe for analysis *via* gas chromatography.

### Desorption experiments of the immobilized dyes under various pH conditions

The organic dye desorption experiments at various pH values were conducted by putting 3DG–organic composites in 10 mL of DI water. The pH of the solution was adjusted with concentrated hydrochloric acid or 10 M NaOH solution. After being left to stand for 30 min, the solution was analyzed on a Varian ultraviolet-visible light (UV-vis) spectrophotometer (Cary-50, Varian Co.) to determine the amount of desorbed organic dyes.

### Adsorptivity measurements

The adsorptivity measurements of the 3DG–EY-1.5 composites towards 4-NA were performed in a manner similar to the photocatalytic activity tests in the dark for 1 h and the concentrations of 4-NA or Cr(vi) were analyzed using a Varian ultraviolet-visible light (UV-vis) spectrophotometer (Cary-50, Varian Co.).

### Probing of the spatial reactive sites

The determination of the spatial reactive sites of the 3DG–organic photocatalysts was performed *via* a procedure similar to the photocatalytic hydrogenation of 4-NA, where AgNO_3_ (the concentration of Ag^+^ was 10 ppm) was used to replace 4-NA and L-3DG–EY-1.5 was the photocatalyst. After the visible light irradiation, L-3DG–EY-1.5 was rinsed with water and freeze-dried for further characterization.

## Conflicts of interest

There are no conflicts to declare.

## Supplementary Material

Supplementary information
