# Supplementary material for: An adaptive geometry regulation strategy for 3D graphene materials: towards advanced hybrid photocatalysts
Source: Chem Sci. 2018 Sep 24;9(47):8876–82. doi: 10.1039/c8sc03679a (PMC6296298; doi:10.1039/c8sc03679a)
Supplement: Supplementary file 1 [file SC-009-C8SC03679A-s001.pdf]

Electronic Supplementary Material (ESI) for Chemical Science.
This journal is © The Royal Society of Chemistry 2018

Electronic Supplementary Information

Adaptive geometry regulation strategy for 3D graphene materials: towards advanced hybrid photocatalysts

Xiuqiang Xie,[ab] Nan Zhang,[ab] Zi-Rong Tang,[b] and Yi-Jun Xu*[ab]

[a] State Key Laboratory of Photocatalysis on Energy and Environment, College of Chemistry, Fuzhou University, Fuzhou 350116, P. R. China

[b] College of Chemistry, New Campus, Fuzhou University, Fuzhou 350116, P. R. China

E-mail: yjxu@fzu.edu.cn

Contents list

Fig. S1 Raman spectra of S-3DG, M-3DG, and L-3DG.

Fig. S2 Structures of EY and RB.

Fig. S3 SEM images of S-3DG-EY (a and b) and M-3DG-EY (c and d).

Fig. S4 EDX spectrum of L-3DG-EY.

Fig. S5 Photos of the setup for the photocatalytic activity tests.

Fig. S6 The formula for photocatalytic conversion of 4-NA to 4-PDA (a). UV-vis spectra of photocatalytic conversion of 4-NA over L-3DG-EY-1.5 (b).

Fig. S7 Photocatalytic conversion of 4-NA and Cr(VI) over S-3DG, M-3DG, and L-3DG under visible light irradiation ($\lambda \geq 420$ nm) for 30 min.

Fig. S8 Resistivities of S-3DG-EY-1.5, M-3DG-EY-1.5 and L-3DG-EY-1.5 at a pressure of 12 MPa.

Fig. S9 Photocatalytic H_2 evolution rate in EY solutions over S-3DG, M-3DG, and L-3DG under visible light irradiation ($\lambda \geq 420$ nm).

Fig. S10 Desorption experiments of the immobilized dyes under various pH.

Fig. S11 Photos of mechanical strength of S-3DG-EY-1.5 (a), M-3DG-EY-1.5 (b), and L-3DG-EY-1.5 (c).

Fig. S12 Recycling of S-3DG-EY-1.5 (a), M-3DG-EY-1.5 (b), and L-3DG-EY-1.5 (c) using a tweezer after photocatalytic reactions.

Fig. S13 Regeneration photoactivity test of L-3DG-EY-1.5 for photocatalytic hydrogenation of 4-NA.

Table S1 Geometry parameters of S-3DG-EY, M-3DG-EY, and L-3DG-EY.

Table S2 Maximum immobilized EY and RB over S-3DG, M-3DG, and L-3DG.

Table S3 Results derived from N_2 adsorption-desorption measurements of 3DG-EY-1.5 composites.

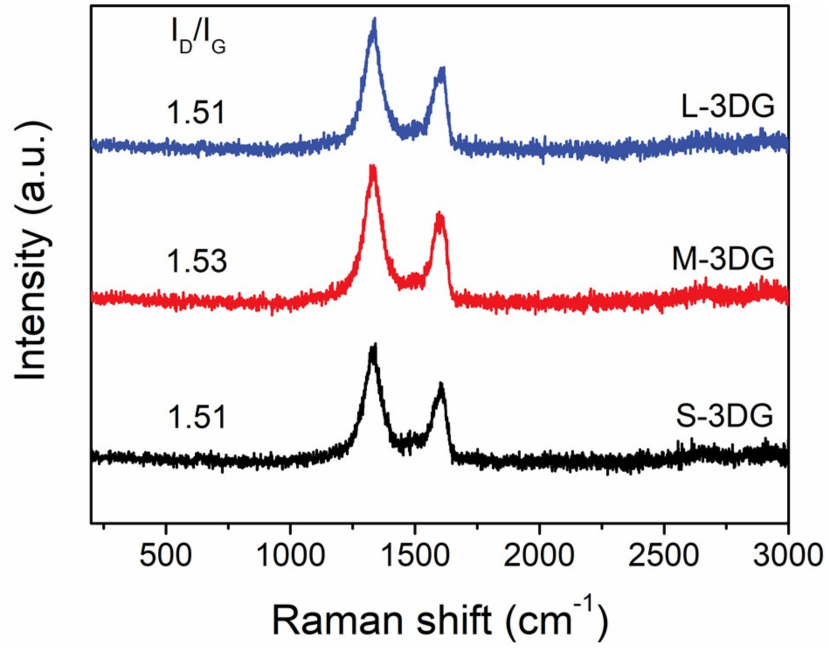

Fig. S1 Raman spectra of S-3DG, M-3DG, and L-3DG.

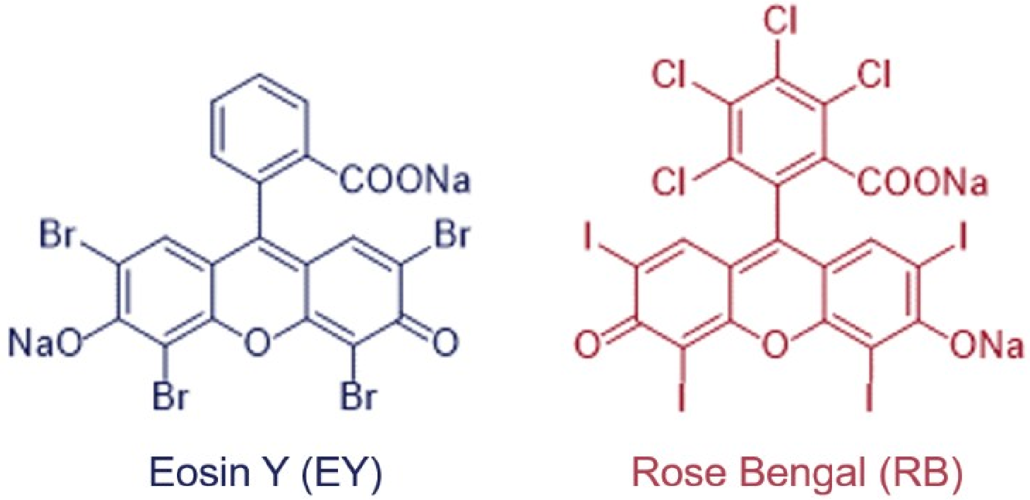

Fig. S2 Structures of EY and RB.

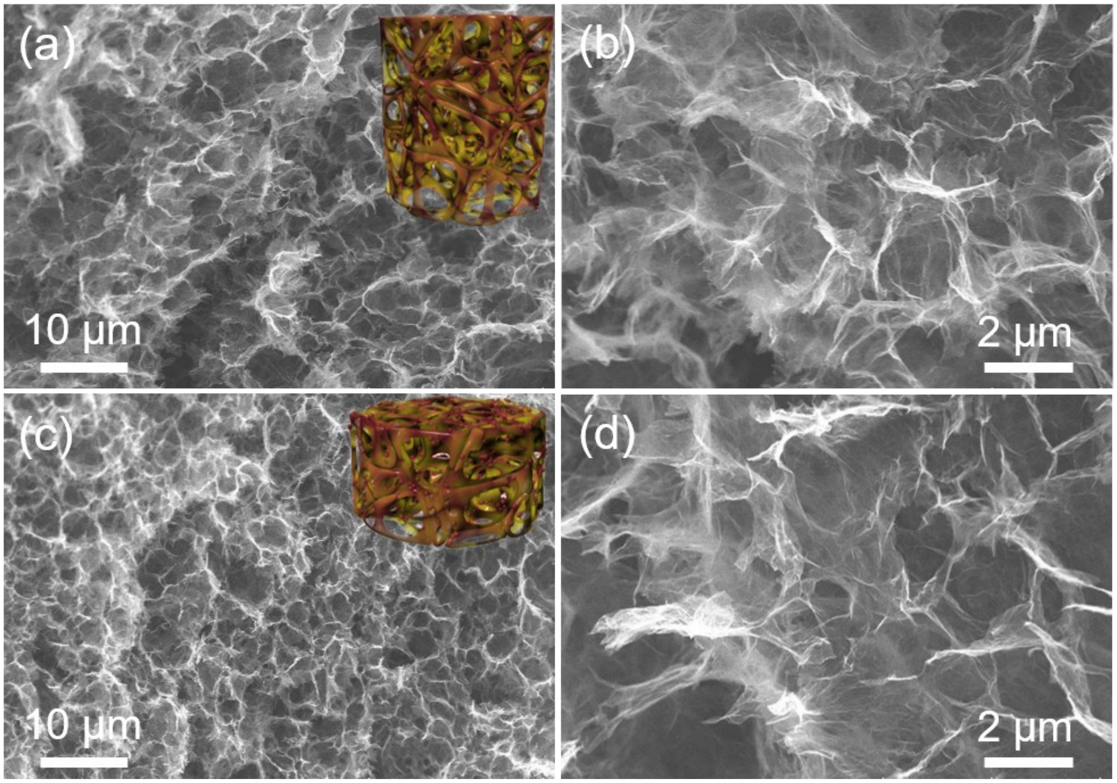

Fig. S3 SEM images of S-3DG-EY (a and b) and M-3DG-EY (c and d).

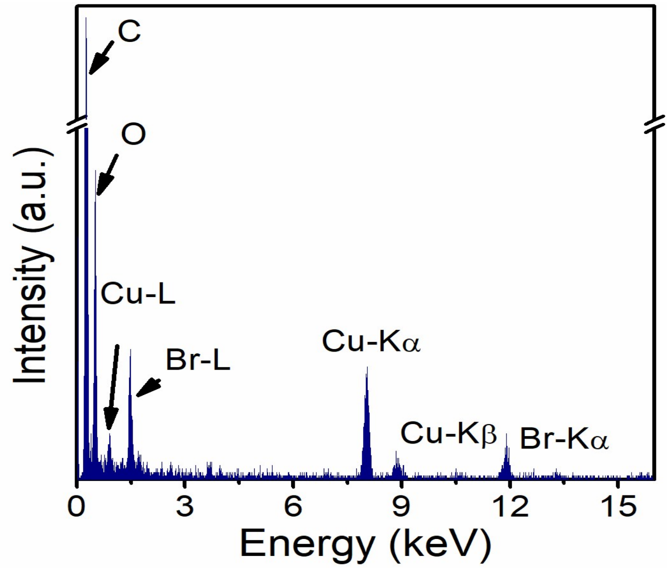

Fig. S4 EDX spectrum of L-3DG-EY.

Note: In addition to Cu signals originating from the Cu mesh as the support for TEM analysis, C, O, and Br signals can be found, which evidences the successful immobilization of EY over 3DG.

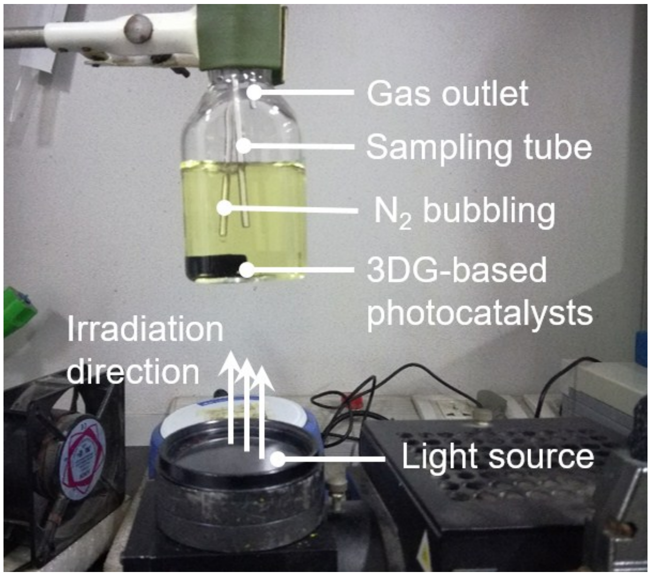

Fig. S5 Photo of the setup for the photocatalytic activity tests.

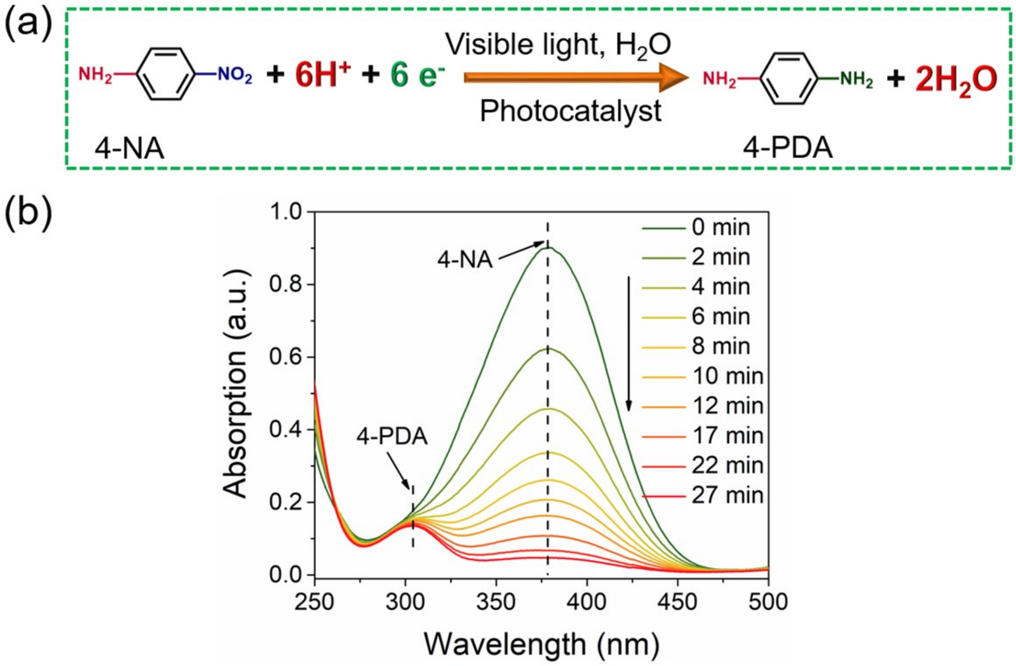

Fig. S6 The formula for photocatalytic conversion of 4-NA to 4-PDA (a). UV-vis spectra of photocatalytic conversion of 4-NA over L-3DG-EY-1.5 (b).

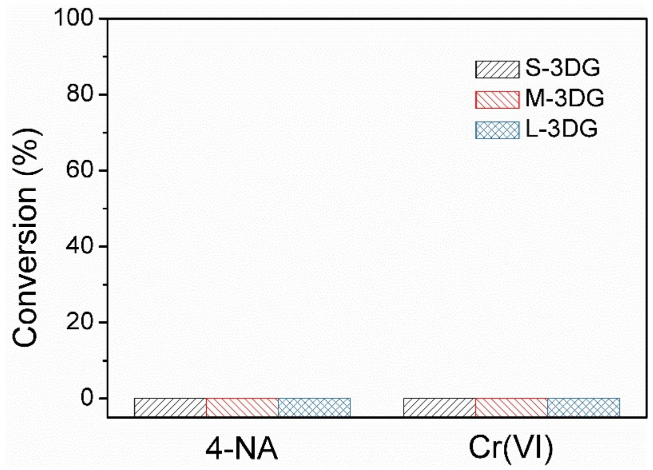

Fig. S7 Photocatalytic conversion of 4-NA and Cr(VI) over S-3DG, M-3DG, and L-3DG under visible light irradiation (λ ≥ 420 nm) for 30 min.

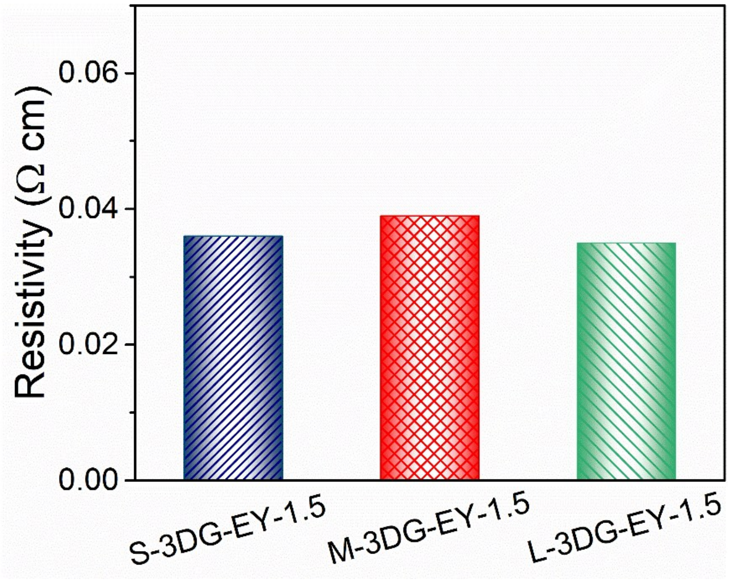

Fig. S8 Resistivities of S-3DG-EY-1.5, M-3DG-EY-1.5 and L-3DG-EY-1.5 at a pressure of 12 MPa.

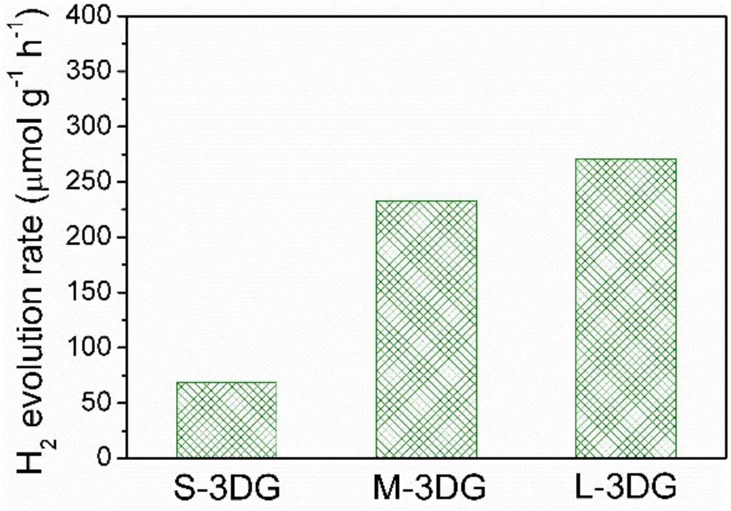

Fig. S9 Photocatalytic H_2 evolution rate in EY solutions over S-3DG, M-3DG, and L-3DG under visible light irradiation ($\lambda \geq 420$ nm).

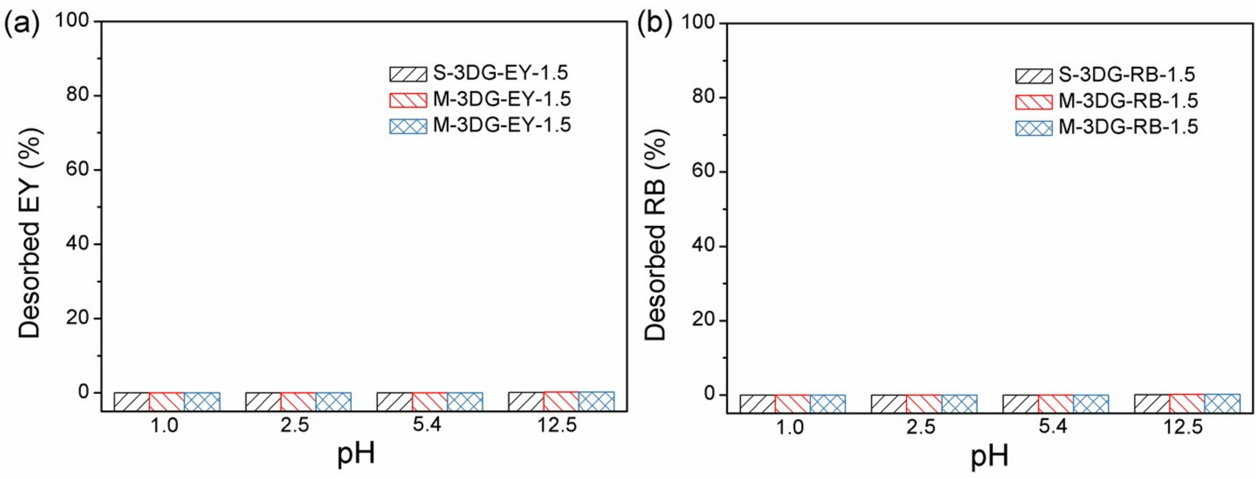

Fig. S10 Desorption experiments of the immobilized dyes under various pH.

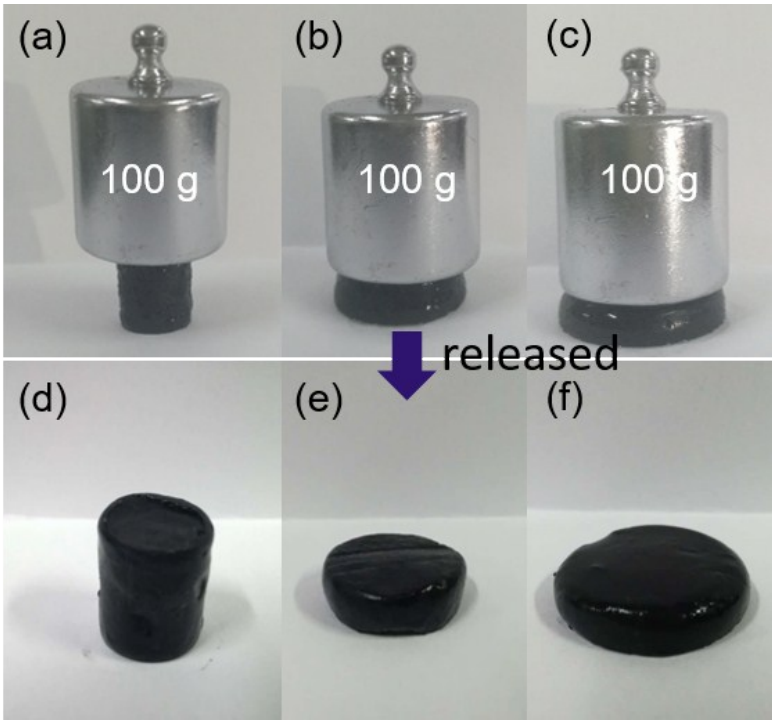

Fig. S11 Photos of mechanical strength of S-3DG-EY-1.5 (a and d), M-3DG-EY-1.5 (b and e), and L-3DG-EY-1.5 (c and f).

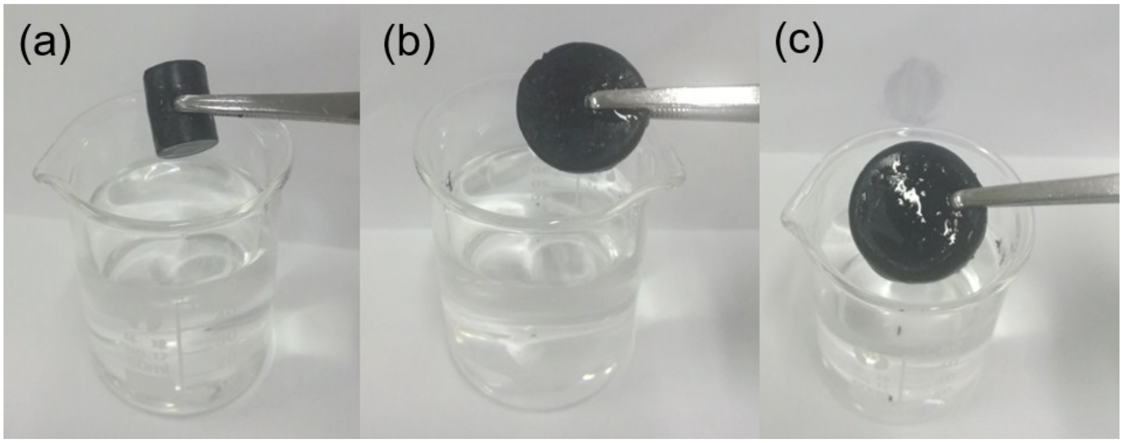

Fig. S12 Recycling of S-3DG-EY-1.5 (a), M-3DG-EY-1.5 (b), and L-3DG-EY-1.5 (c) using a tweezer after photocatalytic reactions.

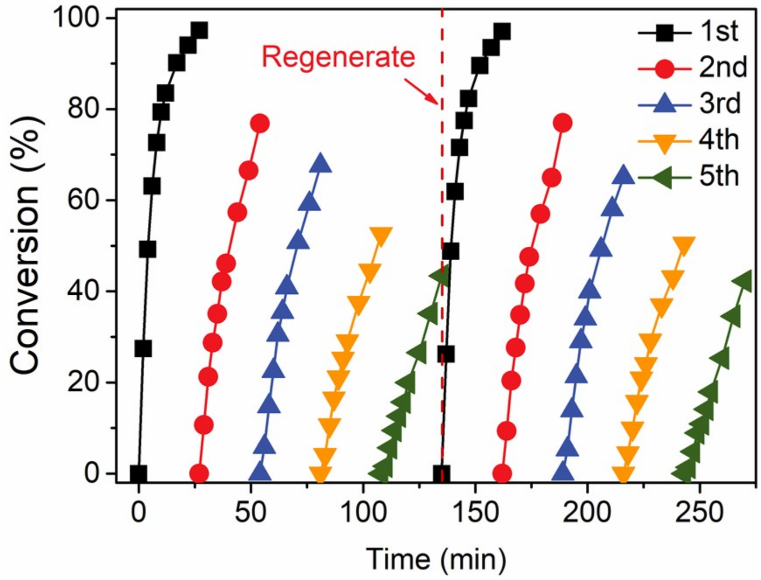

Fig. S13 Regeneration photoactivity test of L-3DG-EY-1.5 for photocatalytic hydrogenation of 4-NA.

Table S1 Geometry parameters of S-3DG-EY, M-3DG-EY, and L-3DG-EY.

Geometry parameter	S-3DG-EY	M-3DG-EY	L-3DG-EY
Diameter (cm)	0.90	1.50	1.82
Height (cm)	2	0.76	0.5
Cross-sectional area (cm^2)	0.63	1.77	2.60
Surface area (cm^2)	4.79	9.85	12.63
Volume (cm^3)	1.26	1.34	1.30
Aspect ratio (diameter-to-height ratio)	0.45	1.97	3.64
Surface-to-volume ratio	3.81	7.34	9.71

Table S2 Maximum immobilized EY and RB over S-3DG, M-3DG, and L-3DG.

Sample	Maximum immobilized EY (mg)	Maximum immobilized RB (mg)
S-3DG	2.62	2.66
M-3DG	2.56	2.60
L-3DG	2.65	2.63

Table S3 Results derived from N_2 adsorption-desorption measurements of 3DG-EY-1.5 composites.

Sample	S_{BET} ($m^2\ g^{-1}$)
S-3DG-EY-1.5	84
M-3DG-EY-1.5	82
L-3DG-EY-1.5	80
